# Pan-hypoileism: an endocrine disease

**DOI:** 10.31744/einstein_journal/2026RW2548

**Published:** 2026-09-23

**Authors:** Sérgio Santoro

**Affiliations:** 1 Hospital Israelita Albert Einstein São Paulo SP Brazil Hospital Israelita Albert Einstein, São Paulo, SP, Brazil.

**Keywords:** Metabolic syndrome, Glucagon-like peptide 1, Peptide YY, FGF19, *Diabetes mellitus*, type 2, Insulin resistance, Gluconeogenesis, Energy metabolism, Obesity, Glucose

## Abstract

Over the past four decades, the gastrointestinal tract has been increasingly recognized as a major endocrine organ. Each of its segments plays a distinct role in digestion, absorption, inter-organ signaling, and metabolic regulation, and each category of food requires a specific physiological response. Consequently, carnivores, herbivores, and omnivores have adapted their digestive systems to meet the demands of their typical diets. Signals arising from the digestive tract influence nearly all other organs and their metabolic activities. Gluconeogenesis, insulin resistance, energy expenditure, the hunger-satiety cycle, glucose effectiveness, lipid clearance, and gastric emptying are examples of metabolic processes profoundly regulated by the gut. Emerging evidence now suggests that the distal part of the human small intestine, the ileum, is progressively failing to perform its full range of functions, including its endocrine roles, in a substantial proportion of the population. This phenomenon may constitute a growing pandemic. The term pan-hypoileism has not previously been used. In this review, we explore why this term deserves recognition as an endocrine disease. Clarifying this condition may be important for both patients and physicians.

## INTRODUCTION

Over the past decades, the gastrointestinal tract has been increasingly recognized as a major endocrine organ.^(1)^ Each of its segments plays a distinct role in digestion, absorption, inter-organ signaling, and metabolic regulation, while different types of food require specific physiological responses. Consequently, carnivores, herbivores, and omnivores have adapted their digestive systems to the demands of their typical diet.^(2)^ Signals arising from the digestive tract influence nearly all other organs and their metabolic activities. Gluconeogenesis, insulin resistance, energy expenditure, the hunger-satiety cycle, glucose effectiveness (GE), lipid clearance, and gastric emptying are controlled to a great extent by gut signals.^(3-5)^

Diabetes, obesity, dyslipidemia, and other elements of metabolic syndrome have become increasingly prevalent,^(6)^ and many factors contribute to these developments. One of these factors, probably a very important one, is the reduced activation (i,e., reduced participation in nutrient absorption) of the distal gut, which has profound metabolic implications.^(3)^

### Gluconeogenesis

Cats are obligate carnivores, and their diet contains only negligible amounts of carbohydrates. Nonetheless, their blood glucose remains tightly regulated within the normal range. Gluconeogenesis is the central metabolic pathway responsible for endogenous glucose production, using amino acids, lactate, and glycerol as substrates.^(7)^

During feeding, a physiological rise in insulin secretion is expected, even if only protein and fat were taken. However, hypoglycemia does not occur because gluconeogenesis is simultaneously upregulated, drawing on dietary substrates to generate sufficient glucose to meet cellular uptake and maintain stable glycemia.^(8)^

How is this process regulated? K enteroendocrine cells (K cells) are dispersed throughout the small intestinal mucosa, where they secrete glucose-dependent insulinotropic peptide (GIP). Glucose-dependent insulinotropic peptide, a gastrointestinal hormone, can stimulate the secretion of both insulin and glucagon.^(9)^ In cats, however, GIP does not respond to oral glucose.^(10)^ Whereas glucagon promotes gluconeogenesis, insulin facilitates glucose uptake, and the balance between these processes minimizes fluctuations in blood glucose levels.^(11)^

Because cats do not consume sugars, gluconeogenesis must operate continuously.^(11)^ Its activity increases during feeding and, naturally, decreases during fasting, when no dietary substrates are available to fuel gluconeogenesis. During these fasting periods, endogenous glucose production (EGP) must be sustained by mobilizing stored gluconeogenic substrates.^(12)^

Accordingly, K cells are found throughout the feline intestinal tract^(13)^ and are markedly more abundant than those found in noncarnivorous species. In cats, GIP-expressing K cells are commonly found from the duodenum to the colon.^(13)^

Humans, like other omnivores, cannot rely on this metabolic strategy because they ingest vegetables that contain carbohydrates. The simultaneous absorption of sugars and activation of gluconeogenesis can lead to hyperglycemia. Consequently, in species that consume carbohydrates, gluconeogenesis must eventually be reduced or suppressed.^(14)^

In nature, plant cells contain substantial amounts of sugars, but these are enclosed within cellulose walls. Cellulose is itself a carbohydrate, but it is extremely difficult to digest. Animals, including humans, require symbiotic bacteria to break it down. Nonetheless, modern humans appear to have an even more limited capacity to digest cellulose than their ancestors.^(15)^

At the beginning of meals containing vegetables, immediate and substantial absorption of sugars is not possible because absorbable carbohydrates are not freely available in nature; food must first be processed. Consequently, the proximal gut of omnivores also contains a substantial number of K cells, similar to the number found in the proximal gut of carnivores,^(16)^ because insulin secretion must occur alongside glucagon secretion to initiate and sustain gluconeogenesis. However, as digestion progresses, simple sugars become available and are increasingly absorbed, at which point gluconeogenesis must be downregulated,^(17)^ unlike in strict carnivores.

The distal segments of the small intestine, particularly the ileum, contribute to the reduction or suppression of gluconeogenesis. Unlike in carnivores, the ileal mucosa of omnivorous species contains a predominance of L cells.^(18)^ L cells produce several peptides and signaling molecules, among which glucagon-like peptide 1 (GLP-1) plays an important role in the suppression of gluconeogenesis.^(17)^

In obesity,^(19)^ type 2 *diabetes mellitus* (T2DM),^(20)^ and metabolic syndrome^(21)^ GLP-1 secretion is generally diminished. Consequently, gluconeogenesis may be inadequately attenuated.^(22)^ Gluconeogenic activity progressively increases as body weight rises.^(23)^ Initially, a compensatory reduction in glycogenolysis maintains total endogenous glucose production (EGP) within the normal range.^(24)^ Later, the increase in gluconeogenesis is no longer adequately offset by a reduction in glycogenolysis; EGP increases, and T2DM may develop.^(23)^

Ileal signaling is deeply involved in the regulation of gluconeogenesis. In the modern era, humans increasingly consume high-glycemic-index foods and ultra-processed foods (HGIFs and UPFs). These foods are evolutionarily abnormal foods for humans and other primates because they contain large amounts of rapidly absorbable simple sugars. Under such circumstances, gluconeogenesis would need to be suppressed rapidly.^(24)^

However, such dietary patterns have become widespread only within the past seven decades, providing insufficient time for meaningful evolutionary anatomical or physiological adaptations. Over thousands of years, evolution shaped the digestive tracts of animals according to their habitual diet, including the digestive tracts of species that consume natural carbohydrate sources. The more nutrient-rich the diet (i.e., lower in cellulose and higher in simple sugars), the easier nutrients are to absorb, and the shorter the small intestine tends to be, consequently reducing the distance between the stomach and the ileum in species adapted to such diets.^(2,25)^ This pattern is observed across vertebrates, from fish to mammals. The length of the small bowel, the types of enteroendocrine cells it contains, and their distribution are tightly linked to the characteristics of the diet.^(2,13)^

The emergence of HGIFs and UPFs has been too abrupt for evolutionary adaptation. In humans, the ileum is now effectively “too distant”: it is stimulated only after substantial amounts of simple sugars have already been absorbed, leading to pronounced postprandial glucose peaks followed by excessive insulin secretion. In this setting, the suppression of gluconeogenesis occurs too late and is insufficient. A young pancreas may compensate by increasing insulin production^(26)^ but obesity and insulin resistance commonly develop over time.^(27,28)^

Under modern dietary patterns, the ileum increasingly fails to suppress gluconeogenesis at the appropriate time and with adequate intensity.

### Insulin sensitivity

Insulin resistance is an evolutionary adaptation.^(29)^ During starvation, animals internalize insulindependent glucose transporters, such as GLUT4, from the plasma membrane into intracellular compartments in tissues throughout much of the body, thereby reducing glucose uptake by these tissues.^(30)^Meanwhile, essential organs, particularly the central nervous system (CNS), rely on insulinindependent glucose transporters such as GLUT3.^(31)^

In this way, the body becomes insulin resistant during periods of scarcity. This is an elegant evolutionary mechanism that preserves glucose for vital tissues, while others depend primarily on free fatty acids (FFA) for energy.^(32)^ Thus physiological insulin resistance serves as a protective adaptation under harsh conditions.

When food is ingested, insulin resistance is initially maintained.^(33)^ However, as more glucose enters the circulation, it becomes both necessary and advantageous to make glucose available to all tissues. Insulin sensitivity then increases through the translocation of glucose transporters such as GLUT4 to the cell surface,^(34)^ this process occurs within minutes.^(35)^

Maximum insulin sensitivity should coincide with the ingestion and digestion of a substantial meal, when the risk of hypoglycemia decreases and hyperglycemia becomes the dominant concern. This enhanced sensitivity is expected when nutrients begin to be absorbed in the more distal sections of the gut – particularly the ileum. Nutrient exposure in the ileum indicates the presence of a large meal, and hormones released from the distal gut are largely responsible for increasing insulin sensitivity.^(36-38)^

The incorporation of HGIFs and UPFs into the diet maximizes proximal nutrient absorption while delaying and diminishing ileal endocrine activity. Consequently, the ileum may fail to induce adequate insulin sensitivity and GE, creating a demand for increased insulin secretion.^(39)^

Insulin is a powerful stimulus for the transcription of GLUT4 and its translocation to the plasma membrane.^(40)^ In insulin-resistant individuals with T2DM, both insulin and glucose levels can be simultaneously elevated, yet glucose uptake remains slow because insulin-mediated GLUT4 translocation is impaired owing to defects in key signaling intermediates.^(41)^

Remarkably, insulin sensitivity improves almost immediately after surgical procedures that promote rapid exposure of the ileum to nutrients, such as traditional biliopancreatic diversions or modern transit bipartition procedures. Glycemia and insulin levels fall concurrently, and glycated hemoglobin typically decreases by around 3% within three months, often returning to the normal range without pharmacological support, which may indicate remission of T2DM.^(41,42)^ Although elevated GLP-1 levels associated with these surgeries are known to enhance insulin sensitivity,^(43,44)^ the magnitude and rapidity of this improvement suggest that additional mechanisms remain to be elucidated.

From an evolutionary perspective, it is plausible that omnivores would maximize insulin sensitivity only in the distal segments of the gut. No ancestral diet could reliably provide safe quantities of simple sugars before nutrients reached the distal intestine. The proximal gut is capable of strongly stimulating insulin secretion. Indeed, GIP is a potent postprandial insulin secretagogue,^(45)^ but for the protection of the central nervous system, it is reasonable that only the ileal signals should simultaneously suppress gluconeogenesis and induce high insulin sensitivity.^(46)^

When modern foods (i.e., HGIFs and UPFs) are disproportionately absorbed during the early phases of digestion, proximal signals such as GIP may be exaggerated, as is commonly observed in obesity and metabolic syndrome.^(47)^ Yet the relative insufficiency of distal gut signals^(48,49)^ fails to trigger the necessary increase in insulin sensitivity. Consequently, insulin production rises, potentially contributing to obesity and inflammation,^(50,51)^ but without effectively correcting hyperglycemia. Pharmacological evidence reinforces this concept: proximal gut hormones alone may even impair glucose regulation,^(52,53)^ whereas distal gut hormone analogs are effective, and their benefits can be enhanced when combined with proximal gut hormone analog.^(54)^

In summary, a welltimed and coordinated ileal endocrine response may play an important role in promoting insulin sensitivity. Populations worldwide are becoming progressively more insulin resistant.^(55)^

### Glucose effectiveness

The ileum contributes to postprandial glycemic reduction not only by suppressing gluconeogenesis, stimulating insulin secretion, and enhancing insulin sensitivity, but also by facilitating glucose uptake into peripheral tissues through an insulinindependent mechanism known as GE.^(56)^

Fibroblast growth factor 19 (FGF19) is a gutderived hormone released from the ileal mucosa during bile acid reabsorption.^(57)^ FGF19 promotes the intracellular conversion of glucose to lactate,^(58)^ thereby reducing intracellular glucose concentrations and facilitating further glucose uptake. This mechanism plays a substantial role in glycemic regulation, with approximately 50% of overall glycemic regulation attributed to GE.^(59)^

FGF19 also increases the transcription and translocation of GLUT4 and its translocation to the plasma membrane of pancreatic β cells.^(60)^ Moreover, FGF19 modulates glucose metabolism in hepatic and extrahepatic tissues through the activation of fibroblast growth factor receptors (FGFRs) and β-Klotho, initiating downstream MAPK/ERK signaling. This process results in increased glycogen synthesis and suppression of gluconeogenesis, promoting glucose uptake independently of insulin.^(61,62)^The upregulation and translocation of GLUT4 are essential for cellular glucose entry, particularly under conditions of insulin resistance.^(63)^

In obesity, metabolic syndrome, and T2DM, circulating FGF19 concentrations may be reduced,^(64,65)^ and GE may also be impaired.^(66)^ This combination may contribute to elevated blood glucose levels and increase the demand for insulin secretion by pancreatic βcell.

Thus, the ileum may also fail to adequately promote GE, a key determinant of glucose metabolism.

### Satiety and gastric emptying

The ileum plays a central role in the induction of satiety. At this stage of digestion, nutrients are not only detected but can also be quantified during absorption. Importantly, absorption, rather than the simple presence of nutrients within the ileum, is required to trigger the expected ileal endocrine response.^(67)^ Malabsorption, such as that induced by certain weightloss surgeries, is therefore not an effective strategy for enhancing distalgut hormonal activity; distal absorption is the key driver.^(68,69)^

During the initial phase of a typical meal in a hungry individual, gastric emptying must proceed rapidly,^(70)^ as the body is in a state of relative starvation. Gastric acid reduces the bacterial load in ingested food, breaks larger particles into smaller ones, and adjusts osmolarity.^(71)^ The acidic environment also facilitates the solubilization and chelation of iron, calcium, magnesium, and zinc through their binding to peptides or amino acids, thereby enhancing micronutrient absorption. Intrinsic factor, secreted in the stomach, binds to vitamin B_12_ to form a complex that is subsequently absorbed in the ileum through specific receptors. After this gastric preparation, the food is ready to progress through the gastrointestinal tract.^(72)^

The stomach rapidly transforms a “general diet” into an “optimized enteral diet”, thereby facilitating absorption. However, because no meaningful absorption occurs in the stomach, nutrients must progress distally; otherwise, the state of starvation persists. Surgical interventions that remove or exclude substantial portions of the stomach, and thereby compromising its functions, place an additional burden on the intestine. The greater the functional loss of the stomach, the greater the intestinal length and capacity required to compensate for this loss and prevent malabsorption.^(73)^

As a meal progresses, the small intestine becomes increasingly occupied. A natural “Paleolithic diet” is difficult to digest, requiring both time and energy. Once the ileum reaches capacity, it activates the socalled “ileal brake”: distal gut hormones, principally GLP-1 and PYY, markedly slow gastrointestinal transit and gastric emptying, allowing sufficient time for digestion and absorption.^(74,75)^

The rise in distal gut hormones and the consequent slowing of transit give rise to the first wave of satiety – intestinal satiety.^(69)^ These hormones can substantially diminish the desire to continue eating.^(76)^

However, humans evolved in environments characterized by prolonged periods of scarcity. Prematurely stopping a meal and leaving valuable food unconsumed would have been disadvantageous. The capacity to store food confers an evolutionary advantage under such conditions. In addition to slowing gastric emptying, GLP-1 promotes relaxation of the gastric fundus,^(75)^ enabling the stomach to accommodate more food and allowing feeding to continue until the stomach is distended. This mechanical response constitutes gastric satiety, which complements hormonally mediated intestinal satiety.^(69)^

Physiologically, hormonal intestinal satiety should precede mechanical gastric distension. Intragastric balloons, used in individuals with obesity to induce weight loss, represent an attempt to reduce food intake.^(77)^ However, a balloon is effectively a foreign body that induces mechanical gastric distension rather than reproducing the physiological endocrine mechanisms of satiety. Moreover, in the absence of elevated distal gut hormone levels, which may indicate both the presence and adequate absorption of nutrients in the distal intestine, gastric balloons, despite causing discomfort, may not provide sustained resolution of obesity and its associated metabolic disturbances. Obesity is fundamentally a complex endocrine and metabolic disorder, not solely a mechanical condition.^(76)^

The secretion of distal gut hormones, including GLP-1, FGF19, PYY, and oxyntomodulin, in response to nutrient absorption in the distal intestine, plays an important role in promoting satiety.^(64,65,76,78)^

In modern populations, this ileal activity may also be blunted, while the prevalence of obesity continues to rise.^(79)^

### Energy expenditure

Low circulating FGF19 concentrations, as observed in T2DM and visceral adiposity,^(64)^ have been associated with reduced energy expenditure and the pathogenesis of obesity.^(80-82)^ Central FGF19 signaling within the hypothalamus enhances sympathetic nervous system activity, which, in turn, stimulates thermogenesis, primarily in brown adipose tissue, thereby increasing energy expenditure and contributing to weight loss.^(82)^ Experimental models show that central administration of FGF19 reduces body weight, improves glucose–insulin homeostasis, and increases the expression of thermogenic genes in adipose tissue.^(58,82)^ These effects are abolished when sympathetic activity is suppressed.^(81)^

FGF19 contributes to reductions in adiposity without necessarily decreasing food intake.^(82)^

Intriguingly, FGF19 is not produced by enteroendocrine cells. Instead, it is synthesized by the ileal mucosa during bile acid reabsorption. Bile acids are predominantly reabsorbed in the ileum, where the mucosa expresses the apical sodium- dependent bile acid transporter (ASBT).^(80)^Low FGF19 levels therefore may support the concept of a broader ileal endocrine insufficiency.

### Blood lipids and systemic arterial blood pressure

FGF19 is a key regulator of cholesterol homeostasis. Low FGF19 levels may increase intestinal expression of NPC1L1 (Niemann-Pick C1-Like 1), a major cholesterol transporter, resulting in enhanced cholesterol absorption and elevated circulating cholesterol levels. FGF19 also promotes hepatic cholesterol efflux through the upregulation of ABCA1 (ATP-binding cassette transporter A1) and other transporters, facilitating HDL biogenesis and transhepatic cholesterol export. Accordingly, reduced FGF19 levels may impair these processes and may be associated with lower HDL-cholesterol levels and higher total cholesterol levels.^(80,83)^ Low FGF19 concentrations are associated with both the presence and severity of coronary artery disease, reflecting a broader association between diminished FGF19 levels and atherogenic lipid profiles.^(84)^

Regarding triglycerides (TGs), low levels of GLP-1, PYY, and FGF19 are associated with elevated TG levels. One of the major metabolic actions of FGF19 is the suppression of hepatic lipid synthesis.^(85)^ This effect is mediated through SHP (SH2 domain-containing protein tyrosine phosphatase) signaling and epigenetic modulation involving DNMT3A (DNA methyltransferase 3A), which together downregulate transcriptional programs responsible for fatty acid production. In the presence of FGF19 deficiency or resistance, as may occur in obesity and nonalcoholic fatty liver disease (NAFLD), this repression is lost.^(86)^ This may result in increased hepatic lipogenesis and elevated circulating triglyceride levels. Experimental models show that the administration of FGF19 suppresses hepatic fatty acid and triglyceride synthesis.^(85)^

GLP-1 also exerts beneficial effects on lipid metabolism. It stimulates hepatic FGF21 expression, which is required for the triglyceridelowering and hepatic lipidregulatory effects of GLP-1 receptor agonists.^(87)^ In models of highfat diet–induced obesity, GLP-1 agonism reduces plasma triglyceride levels, and this effect is lost in the absence of hepatic FGF21,^(88)^ indicating that low GLP-1 activity may contribute to hypertriglyceridemia through impaired FGF21-mediated hepatic lipid regulation.^(89)^

A reduction in ileal endocrine products may therefore affect lipid metabolism and lead to dyslipidemia. High circulating triglyceride concentrations are associated with increased systolic and diastolic blood pressure, as demonstrated by genetic, observational, and crosssectional studies.^(90,91)^ Among individuals with metabolic syndrome, those with hypertriglyceridemia exhibit higher mean arterial pressure than those without hypertriglyceridemia.^(92)^

Accordingly, elevated triglyceride levels may influence blood pressure through mechanisms involving vascular stiffness and inflammatory mediators. GLP-1 is effective in reducing postprandial triglyceride levels.^(93)^

GLP-1 receptors are expressed in the hypothalamus, brainstem, carotid body, vasculature, and kidneys, all of which are involved in blood pressure regulation. Renal GLP-1 receptor activation reduces sodium reabsorption, leading to natriuresis and reduced intravascular volume, which contributes to lower blood pressure. Additionally, GLP-1 suppresses intrarenal angiotensin II production and improves insulin sensitivity, both of which may further contribute to reductions in blood pressure.^(94)^

Observational studies in healthy adults show that higher endogenous GLP-1 secretory capacity is independently associated with lower systolic blood pressure.^(95)^ Endogenous GLP-1 therefore may play a protective role in the development of hypertension. In experimental models of angiotensin II–induced hypertension, preservation of GLP-1 signaling, through receptor agonists or DPP-4 inhibition, attenuates the hypertensive response.^(96)^

Thus, reduced ileal activity may contribute to systemic arterial hypertension.

### Final thoughts

The widespread introduction of HGIFs and UPFs in recent decades constitutes a major biological and evolutionary event, representing a sudden and profound shift in human dietary patterns that has occurred too rapidly for evolutionary adaptation to take place.^(97,98)^ From an evolutionary perspective, the mismatch between modern dietary patterns and physiological adaptations shaped under ancestral conditions may contribute to the increasing prevalence of obesity, dyslipidemia, systemic arterial hypertension, and T2DM.^(25)^

The ileum performs distinct absorptive and endocrine roles. Because HGIFs and UPFs are absorbed rapidly in the proximal gut, ileal absorption is proportionally reduced. This phenomenon may disrupt gut endocrine regulation.^(99)^ Enhanced proximal absorption may lead to hypertrophy of the proximal bowel, further increasing its absorptive capacity. Under an obesogenic diet, progressive weight gain is accompanied by a decline in GLP-1 and PYY secretion.^(100)^

In this scenario, both the absorptive and endocrine functions of the ileum may be diminished, resulting in a state of pan-hypoileism. This review proposes that this state represents an environmentally driven endocrine disorder with potentially important metabolic consequences. Although it has not previously been described using this term, pan-hypoileism is intended to capture both its origin and its therapeutic implications.

Current medical therapies can provide GLP-1 analog, whose effects may be amplified by other gut hormones. However, ileal products remain central to the proposed mechanism. They are potent and may provide benefits that extend well beyond glycemic control and weight loss.^(54)^ Importantly, these benefits can be achieved without anatomical alterations or digestive exclusion, as it occurs with bariatric procedures; pharmacological agents may instead be administered through injectable or oral formulations, without excluding digestive segments.^(84)^

Equally striking are the immediate effects of direct ileal nutrient stimulation.^(42)^ It has long been known that the ileum produces a wide array of endocrine and neuroactive substances when exposed to nutrients.^(76,101,102)^ New surgical procedures that redirect food to the ileum,^(103)^ thereby enhancing distal absorption without excluding any part of the digestive tract, may achieve outcomes comparable to those of the duodenal switch, one of the most effective conventional bariatric operations.

In modern metabolic surgery, malabsorption has been replaced by distal absorption.^(61)^ Distal absorption counteracts the physiological pattern induced by HGIFs and UPFs, which disproportionately favor proximal absorption.^(104)^

These new procedures offer several potential physiological advantages: they stimulate the ileum directly; they promote the release of endogenous hormones, not analogs, at their native site of production, within the intestine; and they activate neuropods, generating both hormonal and neural signaling. Moreover, these signals are mealdependent, varying according to the quantity and quality of the nutrients ingested.^(67)^Together, these mechanisms may contribute to advances in the medical and surgical treatment of metabolic diseases.

Hypothyroidism is a well-recognized endocrine condition. Physicians and patients readily understand the thyroid as an endocrine gland and its relevance to metabolism. This understanding facilitates both diagnosis and treatment.^(105)^ The same, however, may not yet be true of the endocrine functions of the gut.

## CONCLUSION

Although the gastrointestinal tract has been increasingly recognized as a major endocrine organ, the clinical relevance of its endocrine functions, particularly those of the ileum, may not yet be fully appreciated. Patients, likewise, may remain unaware that many of their metabolic conditions may be associated with dysregulation of intestinal endocrine function. Recognizing pan-hypoileism as a proposed endocrine disorder may help advance understanding of this condition. Doing so may help clarify the concept for patients and clinicians alike and may encourage the academic study of intestinal endocrine physiology, which, even today, receives limited attention at the undergraduate level. The concept proposed in this review suggests that pan-hypoileism represents an environmentally driven endocrine disorder with potentially important implications for modern metabolic health.
